# Autoimmune Schizophrenia? Psychiatric Manifestations of Hashimoto's Encephalitis

**DOI:** 10.7759/cureus.672

**Published:** 2016-07-05

**Authors:** Ali S Haider, Maryam Alam, Ebun Adetutu, Richa Thakur, Caleb Gottlich, Danielle L DeBacker, Lianne Marks

**Affiliations:** 1 Texas A&M College of Medicine; 2 Health Science Center, Scott & White Clinic

**Keywords:** autoimmune, hashimoto's encephalopathy, neuroendocrine, psychosis, steroid-responsive, thyroiditis, hashimoto's encephalitis

## Abstract

Hashimoto’s encephalitis (HE), also known as steroid-responsive encephalopathy associated with autoimmune thyroiditis (SREAT), can be a debilitating manifestation of an autoimmune reaction against the thyroid that is often under-diagnosed primarily due to a lack of definitive diagnostic criteria. This is a case of a 52-year-old woman who has been diagnosed with HE after presenting with recurrent and severe psychosis in conjunction with paranoia and a thyroidopathy. Her symptoms are chronic, having first been documented as presenting 15 years prior and showing progressive exacerbation in both frequency and severity. The patient’s paranoia often manifested as delusions involving family members or close friends and consequently introduced an opportunity for harm to herself and others. She showed great conviction with self-diagnoses that were proven incorrect, resulting in occasional non-compliance. Between episodes, the patient did not show evidence of symptoms. This patient struggled with several incorrect diagnoses and treatments for several years before the correct diagnosis of HE was made and displayed extreme improvement upon corticosteroid administration. This case illustrates the importance of increasing awareness of HE as well as including HE in a differential diagnosis when any patient presents with psychosis and concurrent thyroidopathy. Hashimoto’s encephalitis follows putative characteristics of autoimmune diseases, exhibiting a higher incidence in women as compared to men, presenting with increased titers of autoantibodies, and showing dramatic amelioration when treated with corticosteroids.

## Introduction

Hashimoto’s encephalitis (HE), also termed steroid-responsive encephalopathy associated with autoimmune thyroiditis (SREAT), is a rare autoimmune disease characterized by encephalitis associated with anti-thyroid antibodies that may mimic a variety of other neurologic/psychiatric disorders [1]. HE presents a unique diagnostic challenge; the clinical manifestations of the disease often suggest an infectious etiology, yet patients respond to immunosuppressive therapy [2]. Although thyroid levels may be abnormal in HE patients, most patients are euthyroid and have high circulating levels of anti-thyroid antibodies [3]. Managing this disease with a protean number of possible manifestations aims at immunosuppression, generally with the use of corticosteroids, instead of correcting the abnormal levels of thyroid hormone. Although most patients respond to corticosteroids, given the rarity of this disease, a clear treatment regimen has not yet been established [4]. Additionally, although there is increasing recognition of an autoimmune contribution to psychiatric presentations, they are still often missed [5]. This rare case of a 52-year-old woman with an extensive history of neuropsychiatric episodes illustrates the difficulty in diagnosing and treating a patient with Hashimoto’s encephalitis.

## Case presentation

The patient is a 52-year-old female who suffers from a confirmed case of Hashimoto’s encephalitis after presenting with recurrent psychosis and paranoia in conjunction with her thyroidopathy. Laboratory findings revealed anti-microsomal (TPO) antibody titer of 1:1600 and an anti-thyroglobulin titer of 1:80. This patient’s most recent psychotic episode led to her being placed in emergency detention after delusions of being sprayed with poison and claiming that her family was part of the mob.  

The patient has been suffering from intermittent paranoia, anxiety, and associated psychosis for at least 15 years. At the time of this patient’s evaluation, she was concerned about a rash that occurs associated with these episodes, where she frequently ends up in a mental hospital due to her ‘schizophrenia’ like appearance. The patient believed that this rash was either caused by Porphyria or Lyme disease and had done extensive research as "most doctors just think I’m crazy and don’t believe that there is something actually wrong with me." On further evaluation of her rash including multiple biopsies, clinical appearance, and the patient’s own report; these excoriated-appearing ulcerations ended up being related to neurodermatitis that manifests concurrently with her psychotic episodes. When she has these psychotic episodes, the patient has historically learned to cope by separating herself from others for multiple days. She had one episode where she was arrested due to hiding herself in an abandoned house with a gun. She is usually convinced that others were trying to hurt her during her episodes and has provided multiple stories of how which are usually related to a persecutory delusion, such as poisoning or related to the mob or mafia. There has been no evidence of aggressive behavior by her during her episodes with the exception of some verbal aggression. However, the patient had learned to escape emergency detainment in the hospital during an episode simply by learning to repeat the phrase "I am not a harm to myself or others." The patient's son provided a recorded example where she had just finished a tirade about how she had been undergoing chemical attacks by the mafia where she utilized this phrase to be discharged from a hospital. Often, her family would be unable to locate her during these episodes unless they were contacted by the local police or hospital. On average, these episodes occurred approximately once yearly, but over the course of 15 years, they occurred more frequently as time went on. Her paranoia usually manifested most significantly toward her family members, and one consistency to her episodes was the temporary viewpoint by the patient that her husband was trying to kill or hurt her and that strangers were trying to hypnotize her. Outside of her episodes, she had no complaints about her husband or her safety. Several times, concurrent with these episodes, the patient would start rapidly blinking which she would justify as her attempt to hypnotize others before they were able to hypnotize her. Sometimes this was her son’s first clue that she was starting another episode. It is unclear if these actions were related to any seizure activity. However, the patient has had two EEGs performed, which resulted in normal findings with the exception of increased beta activity. It is clear from this patient case that a patient with an HE flare could potentially be a risk to society and themselves as well as how limited our society is in treating patients with ‘atypical’ mental disorders.

Apparent paranoia and anxiety were frequently evident, and the patient would bring extensive documents she had printed out from the internet. This started with concern over Lyme disease or Porphyria, and after her paraneoplastic antibody came back positive, it switched to concerns over undiagnosed malignancy. Testing for Porphyria was negative as well as for viral illnesses such as HIV and Hepatitis B and C. Lyme antibodies were positive but PCR was negative. She has been previously diagnosed with paraneoplastic syndrome after a positive finding of Anti-Yo antibodies were found. Extensive follow-up tests were run including CT scans, PET scans, MRI, breast mammogram, and pathology after a hysterectomy, all of which yielded negative results for malignancy. A repeat paraneoplastic panel was performed and came back negative, and the first test was suspected to be a false positive. Other testing, including for *Histoplasma*, *Blastomyces*, *Cryptococcus*, *Coccidiodes*, VDRL, ANCA, anti-SSA/SSB was all negative. Also normal were levels of ceruloplasmin, copper, B12, folate, niacin, thiamine, cortisol, Vitamin D, and parathyroid hormone.

The patient has also presented with paranoia related to her medications. Her medical chart indicates that she has a total of 63 drug allergies, which was contradicted by the patient outside of a psychotic episode where her allergies were reviewed and she claimed to only have one allergy, erythromycin, which was not one of the 63 noted in the chart. At several points, the patient became concerned with her prescribed generic levothyroxine as well as her name-brand levothyroxine sodium tablets. She started to obtain compounded levothyroxine, but soon developed a concern that she had an allergy to this as well, after which she was given thyroid tablets, USP. 

More recent tests show a positive ANA (1:160, speckled). An MRI also revealed a small frontal meningioma as well as a chronic lacunar infarct in her right basal ganglia, which are thought to be unrelated to her symptoms. A muscle biopsy showed only minor nonspecific abnormalities while PET and CT scans continued to be negative for malignancy. A stable <4 mm nodule was seen in her right lung apex. TSH showed elevation to 25 during a psychotic episode, with proposed correlation to under-compliance with thyroid medications related to her paranoia. Hepatitis was occasionally seen concurrent with episodes, such as a finding of AST in the high 200’s on one occasion. CSF findings have all been benign. 

Physical exam findings during an episode were significant for paranoid and argumentative and frequently tangential affect as well as hypopigmented patches at sites of previous excoriations from suspected neurodermatitis. The paranoia and psychosis were significantly variable over time, and although the paranoia could exist independently of her psychosis, they were predominantly temporally related--the closer to the psychotic episode, the higher the level of paranoia. Episodes of psychosis also presented with both auditory and visual, and occasionally olfactory hallucinations. 

Individual symptoms undulated over time with correlations previously noted, the most prominent being the flares of neurodermatitis and neuropsychiatric symptoms including paranoia and psychosis. Her primary outpatient psychiatrist decided that the patient’s condition was medical and not psychiatric, primarily because this patient was ‘normal’ between her episodes with the exception of possible increased paranoia/anxiety, and weaned the patient off of her medications. Her temperature could be slightly elevated during an episode to 99-100 degrees Fahrenheit. On occasion, other symptoms would present such as abdominal pain, diarrhea, myalgias, arthralgia of the shoulder, ankles, and lower back, intermittent headaches, olfactory hallucinations, sleep disturbances, anxiety, and at times significant memory loss. Her case was discussed between psychiatry, neurology, and internal medicine; and one gram IV methylprednisolone sodium succinate daily for five days was administered and the patient reported significant improvement and a resolution of her ‘episode’ within one week. On six-month follow-up, the patient was free of psychotic symptoms and functioning well.

## Discussion

Hashimoto’s encephalitis is believed to be under-diagnosed due to its myriad of clinical presentations as well as the lack of definitive diagnostic criteria [6]. Generally, the most common symptoms include sub-acute confusion with additional neurologic symptoms like seizures or changes in consciousness. The mechanism by which HE causes disease is not well understood; it has been proposed that it might be caused by immune complex deposition, vasculitis, or other inflammatory conditions [7]. HE is considered immune-mediated as opposed to complications from abnormal circulating thyroid levels, as disease severity does not typically appear to correlate with thyroid function level [4]. Disease severity also does not always correspond to thyroid antibody titer [8]; however, immunosuppressive therapy does improve HE as well as usually lowers circulating antibody levels. Furthermore, HE presents similar to other autoimmune diseases, including by tending to affect women, with the most common age of onset being in the 40’s, and at a much more frequent rate than men [8-9].

Here, we have presented a case of a 52-year-old female with Hashimoto’s encephalitis who presented predominantly with psychiatric symptoms. Her paranoia and psychosis suggested a neurological or psychiatric origin as opposed to an endocrine or an autoimmune issue. She was frequently diagnosed with schizophrenia during her multiple episodic hospitalizations, particularly due to the frequent lack of other signs of disease. Other causes of rapidly progressing delirium and mental status changes were also considered including strokes, transient ischemic attacks, paraneoplastic syndromes, and metastatic cancer, all of which were negative or insignificant.

Confirmation of Hashimoto’s encephalitis requires elevated titers of antithyroglobulin or anti-thyroid peroxidase antibodies, in addition to the clinical manifestations of the disease [4]. Both titers for this patient were elevated, while other studies were inconclusive. Furthermore, responding to corticosteroids confirms this diagnosis of Hashimoto’s encephalitis. Since Hashimoto’s encephalitis is a rare disease, the current treatment regimen has not been well established. Patients are usually started empirically on corticosteroids [9]. This patient received a five-day course of one gram daily IV methylprednisolone sodium succinate which produced complete resolution of her psychosis. 

## Conclusions

In conclusion, Hashimoto’s encephalitis, first described in 1966, presents a diagnostic conundrum since clinical manifestations frequently suggest either a psychiatric disorder or an infectious etiology [10]. Symptoms usually occur either episodically, as seen in this patient, or with insidious progression along the disease course. However, the treatment must focus on immunosuppression to work effectively. HE is by definition usually responsive to steroids and was dramatically so as seen in this patient [9]. 

When treating a patient presenting with psychotic symptoms, it is important to include HE in the differential diagnosis as well as rule out any other causes of delirium. In fact, Hashimoto’s encephalitis should be considered in all patients who present with an acute or subacute neuropsychiatric disorder of unclear etiology, particularly with current or previous thyroid dysfunction [9]. Finally, the evidence of autoimmune thyroiditis can be separated from neuropsychiatric symptoms by decades, making HE even more of a diagnostic dilemma.
